# Giant Congenital Splenic Cyst in a Pediatric Patient: A Case Report and Review of Surgical Management

**DOI:** 10.7759/cureus.102228

**Published:** 2026-01-24

**Authors:** Qasim Alnahawi, Raja Nadeem, Amena F Almubarak, Jenna A Almannaei, Abdulmenem Abualsel

**Affiliations:** 1 General Surgery, King Hamad University Hospital, Muharraq, BHR

**Keywords:** abdominal cyst, congenital splenic cyst, laparoscopic partial splenectomy, pediatric surgery, splenic preservation

## Abstract

Congenital splenic cysts are rare entities, accounting for approximately 10% of non-parasitic splenic cysts, and are often incidentally discovered. Although many remain asymptomatic, larger lesions may present with abdominal discomfort or complications requiring intervention. We report the case of an 11-year-old boy who presented with chronic abdominal discomfort and back pain and was found on imaging to have a giant splenic cyst measuring 16 × 14.5 cm, located at the upper pole of the spleen with internal echoes and calcifications. The patient underwent a laparoscopic partial splenectomy, during which approximately 1.5 liters of serous fluid were evacuated from the cyst. The postoperative course was uneventful, and the patient was discharged on postoperative day two. Histopathological examination confirmed the diagnosis of a splenic epithelial cyst. Congenital splenic cysts are believed to arise from mesothelial inclusions during embryogenesis. While conservative management may be appropriate for small, asymptomatic lesions, large or symptomatic cysts generally warrant surgical treatment. Spleen-preserving techniques, particularly laparoscopic partial splenectomy, are increasingly favored as they provide symptom relief while maintaining splenic immune function. This case highlights the safety and effectiveness of a minimally invasive, spleen-preserving approach in pediatric patients with giant congenital splenic cysts.

## Introduction

Congenital splenic cysts are rare lesions, accounting for approximately 0.07% of all splenic cysts [1]. They are most commonly identified in children and young adults, with a slightly higher incidence in females. These cysts arise from mesothelial inclusions during embryogenesis and are classified as true cysts due to the presence of an epithelial lining, distinguishing them from post-traumatic pseudocysts, which lack epithelial tissue [2].

The Martin classification categorizes splenic cysts into primary (true) and secondary (pseudocysts) types [3]. True cysts are further subdivided into mesothelial and endothelial subtypes based on histopathological characteristics [4]. Although many congenital splenic cysts remain asymptomatic, larger lesions may cause abdominal pain, early satiety, urinary urgency, or lead to complications such as rupture, hemorrhage, or infection [5].

Surgical intervention is recommended for symptomatic cysts, rapidly enlarging lesions, or those complicated by infection or bleeding. In recent years, minimally invasive, spleen-preserving techniques-particularly laparoscopic partial splenectomy-have gained preference in pediatric patients to reduce postoperative morbidity while maintaining splenic immune function [6].

Because of the rarity of giant congenital splenic epithelial cysts in children and the current revolution towards minimally invasive techniques, documenting such cases adds valuable clinical and technical insights to the existing literature and helps guide optimal surgical decision-making in pediatric patients.

## Case presentation

An 11-year-old medically free boy presented to the pediatric surgery clinic in August 2024 with chronic back pain radiating to the abdomen, progressive abdominal heaviness, and urinary urgency. Symptoms persisted for one year, with worsening discomfort after meals and intermittent constipation. He denied vomiting or respiratory symptoms. In the week before the presentation, the pain intensified and became continuous.

Physical examination revealed stable vital signs and palpable splenomegaly measuring approximately 10-12 cm, with a distinct splenic notch appreciated on inspection and palpation.

Baseline blood tests, including complete blood count (CBC), inflammatory markers, and liver enzymes, were within normal limits.

Imaging

**Figure 1:**
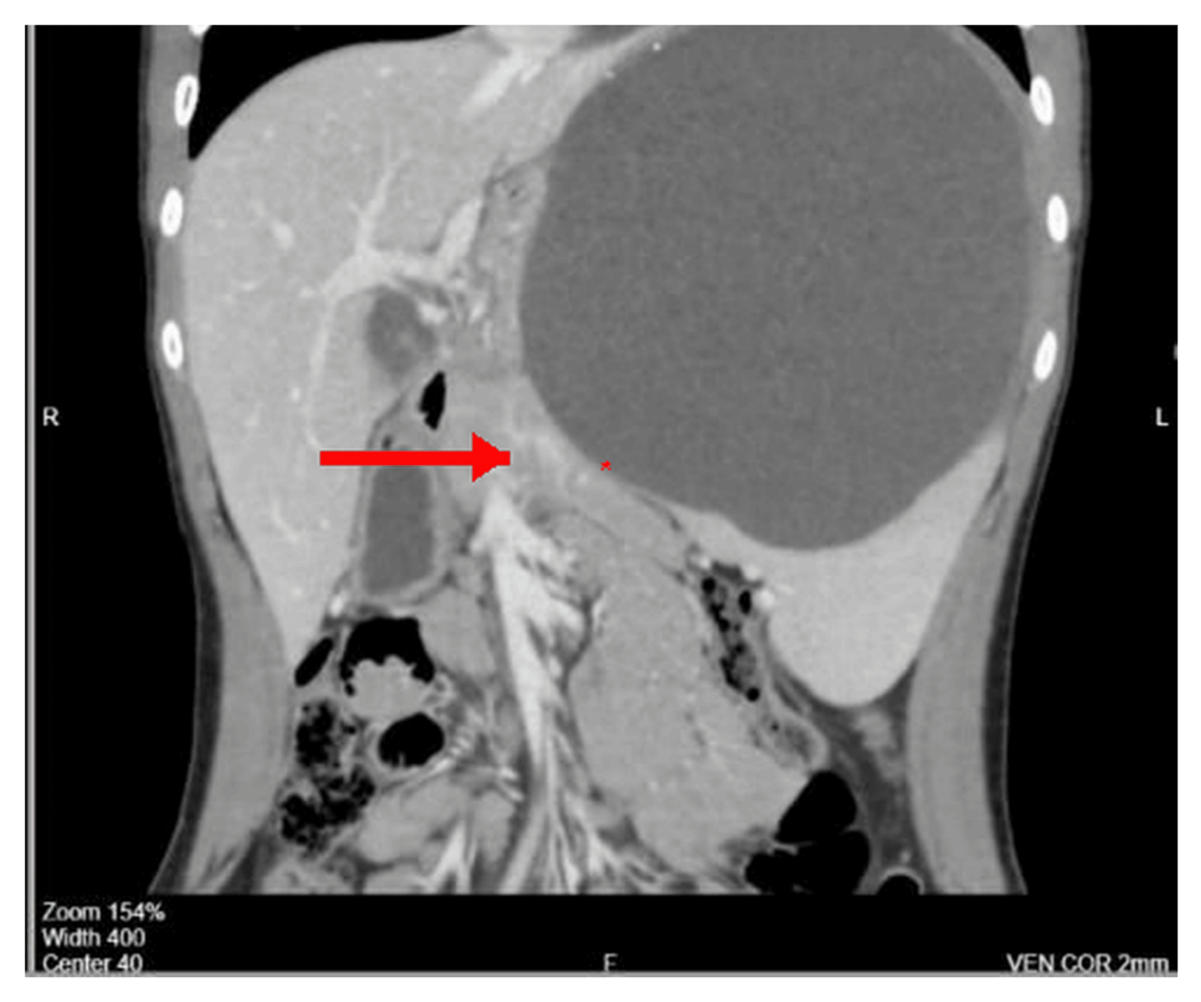
Coronal CT scan showing a large thin-walled splenic cyst occupying the upper pole with peripheral calcifications. Axial contrast-enhanced CT scan of the abdomen showing a giant splenic cyst occupying the upper pole of the spleen, causing displacement of adjacent structures including the stomach and left kidney (asterisk marks cyst cavity).

**Figure 2:**
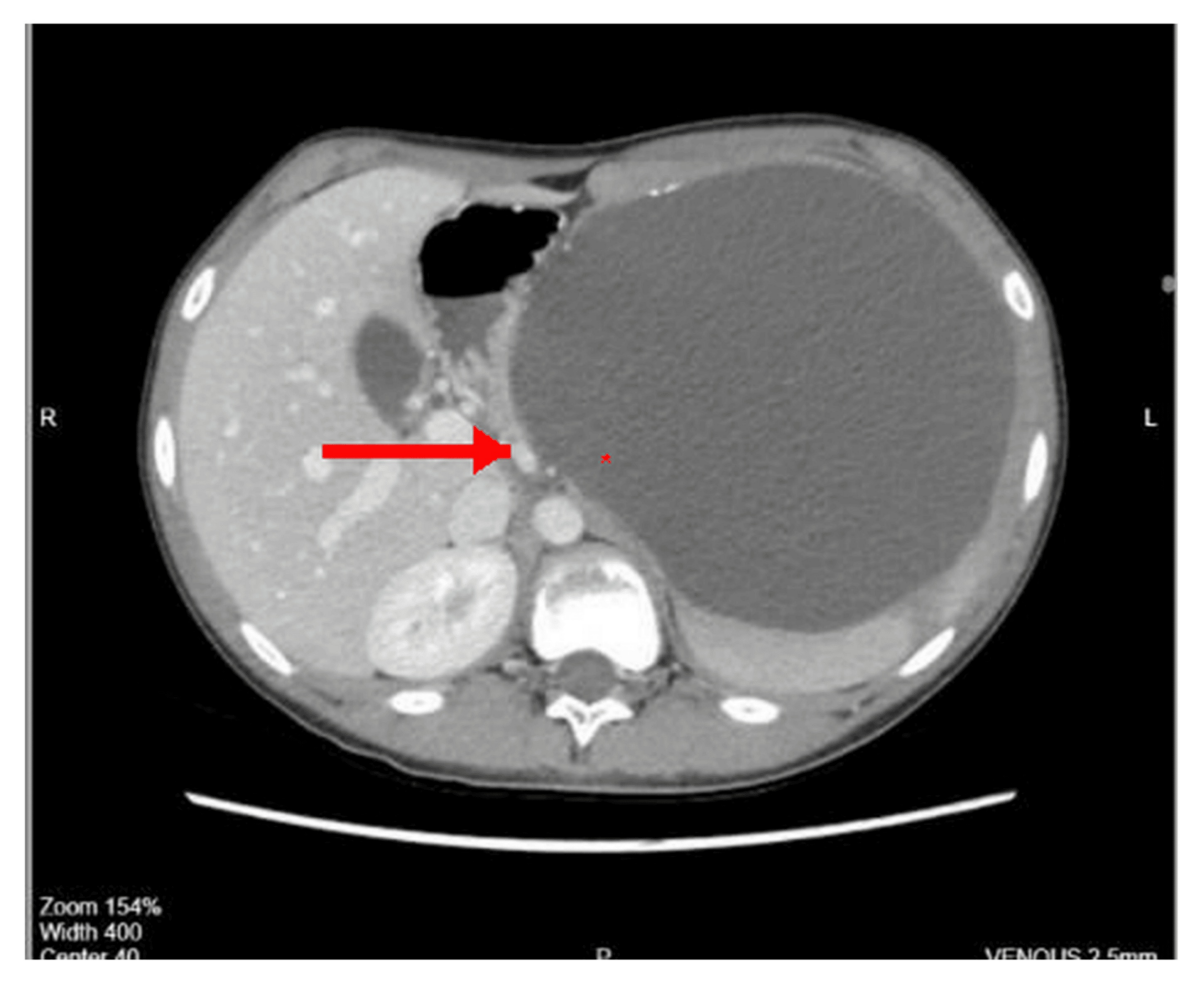
Axial CT scan showing a giant splenic cyst with internal echoes and displacement of adjacent structures. Coronal contrast-enhanced CT image demonstrating a large, well-defined, thin-walled splenic cyst occupying the superior pole with compression of surrounding abdominal organs (asterisk marks cyst cavity).

**Figure 3:**
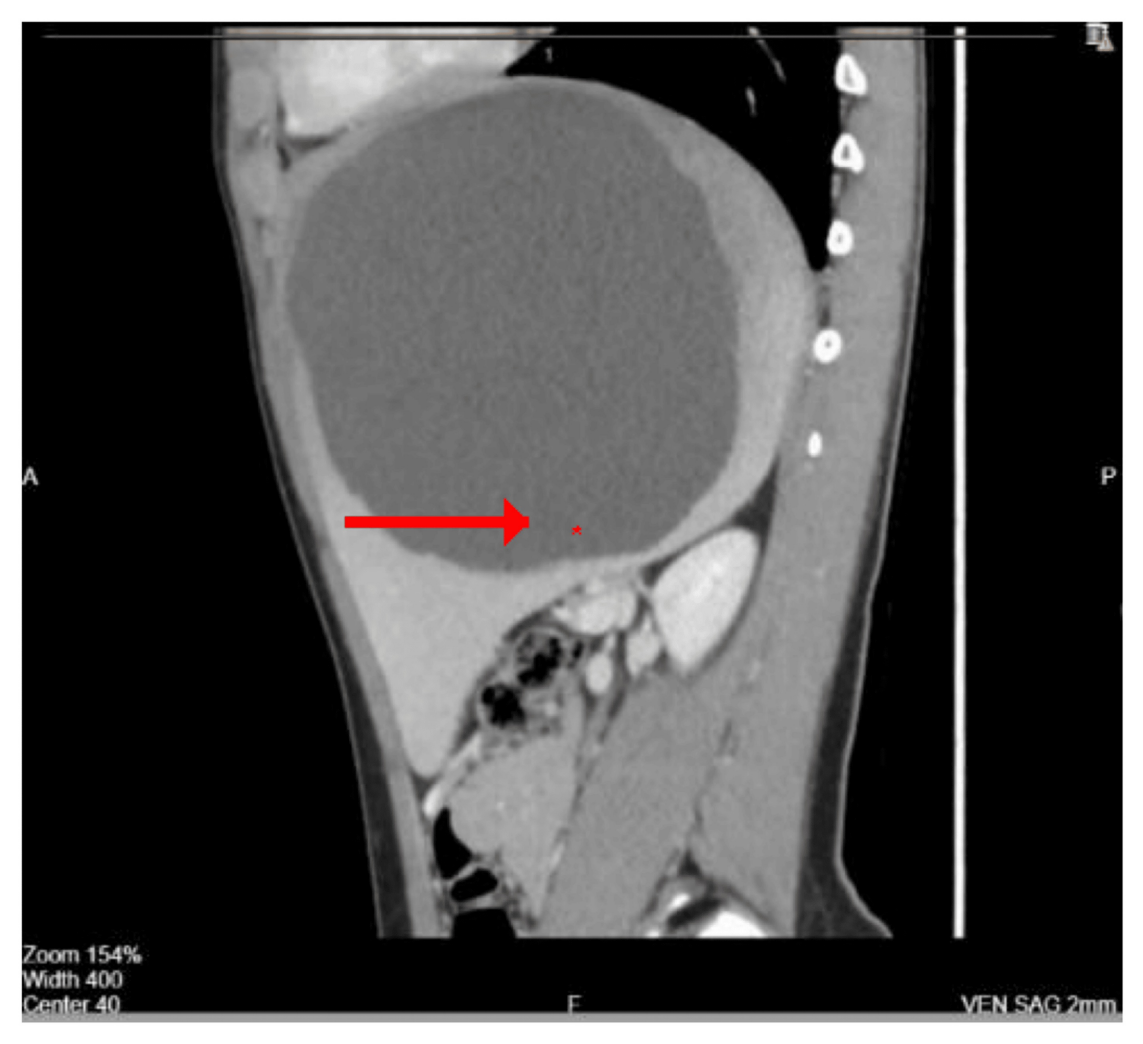
Sagittal CT scan demonstrating the cyst dimensions and its relation to surrounding organs. Sagittal contrast-enhanced CT view revealing the vertical extent of the splenic cyst and its close relationship to the diaphragm and stomach (asterisk marks cyst cavity).

Abdominal ultrasound demonstrated a giant upper-pole splenic cyst measuring 16 × 14.5 cm with internal echoes and turbid contents. Contrast-enhanced CT confirmed a large, thin-walled cyst with peripheral calcifications consistent with a congenital splenic epithelial cyst (Figures 1, 2, 3).

Preoperative preparation

Vaccination against encapsulated organisms was completed, and pediatric hematology clearance was obtained.

Operative procedure

In September 2024, the patient underwent laparoscopic partial splenectomy targeting the upper pole. Pneumoperitoneum was established using a Veress needle infra-umbilically. Four ports were placed: 5 mm infra-umbilical, 12 mm left lumbar, 5 mm right upper quadrant, and 5 mm epigastric.

Intraoperative findings included a large cyst abutting the stomach medially and adherent to the diaphragm and lateral abdominal wall. A total of 1.5 liters of serous cystic fluid was aspirated. The splenic artery branch feeding the upper pole was clipped and divided. The cyst wall and associated upper splenic pole were excised with careful adhesiolysis from the stomach and diaphragm. Residual epithelial lining was cauterized, and an abdominal drain was inserted.

Postoperative course

**Figure 4:**
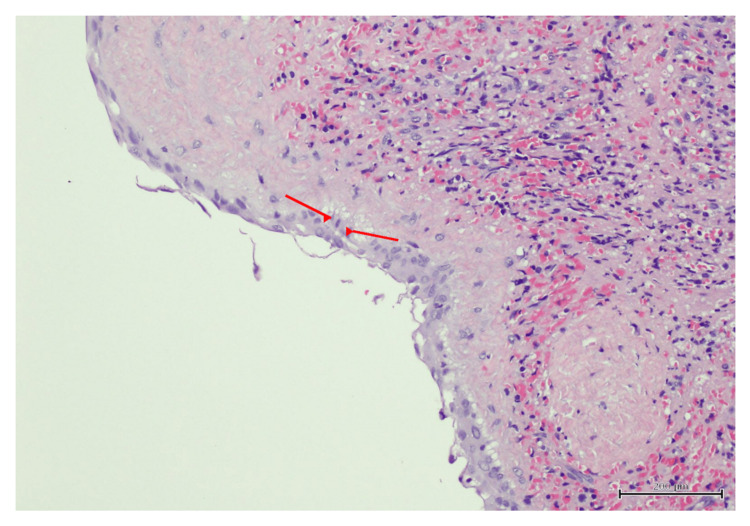
Histopathology of splenic epithelial cyst. Histopathology of the cyst wall (H&E stain, 10× magnification) showing flattened epithelial lining (red arrows) consistent with an epithelial cyst, accompanied by chronic inflammatory cell infiltrate. Scale bar = 200 μm.

**Figure 5:**
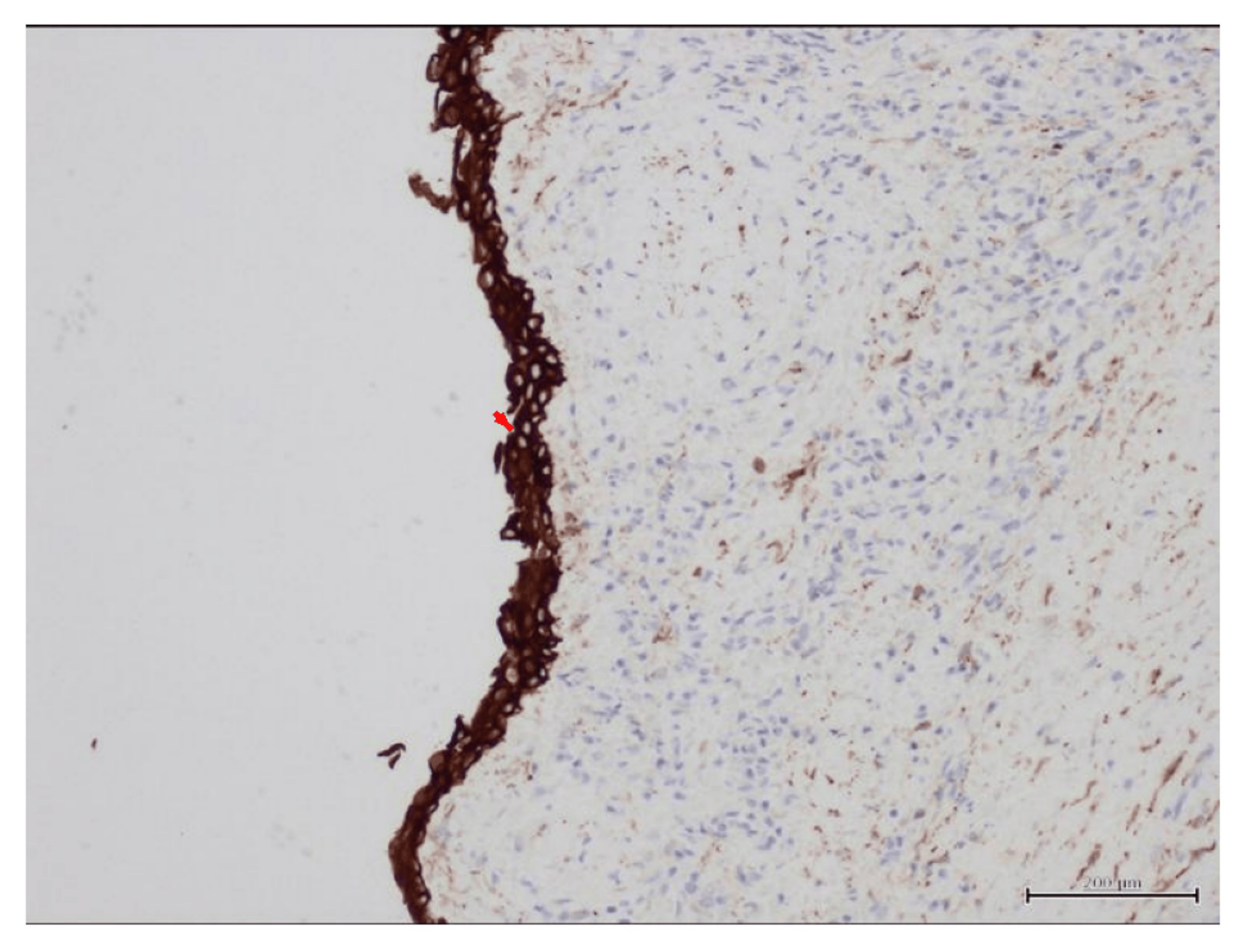
Immunohistochemistry of splenic cyst wall. Immunohistochemistry staining showing strong cytokeratin positivity along the cyst lining (arrow), confirming the epithelial origin of the splenic cyst.

**Figure 6:**
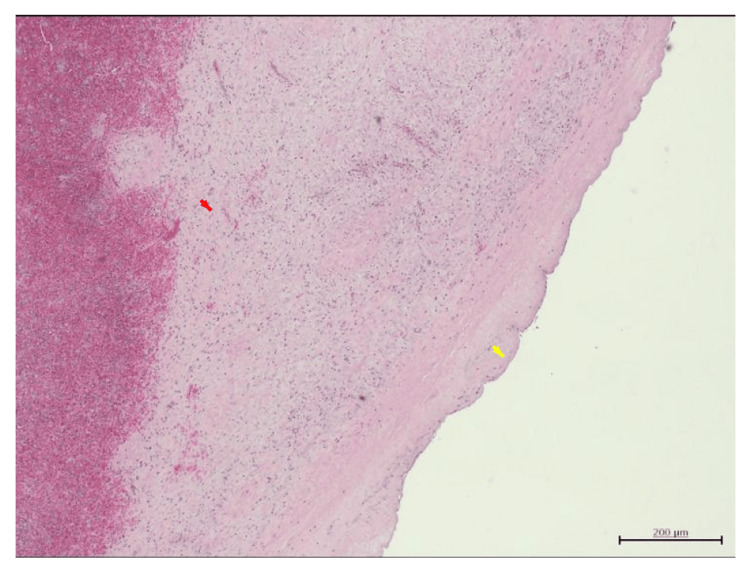
Histopathology of splenic cyst wall (H&E Stain). High-power view demonstrating cuboidal epithelial lining of the cyst (yellow arrow) and dense inflammatory cell infiltrate within the cyst wall (red arrow).

Recovery was uneventful. The drain was removed on postoperative day two, and the patient was discharged on oral antibiotics and analgesics. Histopathology confirmed a benign splenic epithelial cyst, and aspirated fluid analysis was negative for parasitic infection (Figures 4, 5, 6).

At two weeks of follow-up, the patient reported complete resolution of abdominal and back pain, with no urinary urgency or gastrointestinal symptoms. Physical examination shows healed surgical sites with no abdominal tenderness. The patient returned fully to school and daily activities without restrictions, and no postoperative complications were noted during follow-up.

At three-month follow-up, the patient remained asymptomatic with normal appetite, bowel habits, and activity level. Serial laboratory investigations, including complete blood count and inflammatory markers, remained within normal limits in all visits, indicating preserved splenic function.

## Discussion

Congenital splenic cysts are rare developmental lesions arising from mesothelial cell inclusions during embryogenesis, with an epithelial lining that distinguishes them from post-traumatic pseudocysts [1-3]. Consistent with published series, these cysts are most frequently identified in children and young adults and often remain clinically silent until they reach a substantial size [4]. In the present case, delayed presentation with progressive symptoms over one year reflects the indolent growth pattern commonly described in the literature, where diagnosis is often made only after mass effect becomes clinically significant.

The patient’s symptoms-abdominal discomfort, early satiety, constipation, and urinary urgency-are comparable to those reported in cases of giant splenic cysts exceeding 10 cm in diameter [5]. Several authors have emphasized that symptom severity correlates more closely with cyst size and location than with histologic subtype. The upper-pole location in our case likely contributed to diaphragmatic irritation and back pain, a feature reported less frequently but documented in large cysts abutting the diaphragm or posterior abdominal wall. The absence of acute complications such as rupture or infection at presentation contrasts with some reported pediatric cases, underscoring the variability in clinical behavior despite similar cyst dimensions.

Historically, total splenectomy was advocated for large or symptomatic cysts due to concerns regarding recurrence and technical complexity [6]. However, accumulating evidence regarding the spleen’s immunologic role, particularly in pediatric patients, has shifted management toward spleen-preserving strategies [7]. Our surgical approach aligns with current literature favoring laparoscopic partial splenectomy, which has demonstrated low recurrence rates and favorable functional outcomes when complete excision of the cyst wall and devascularization of the affected splenic segment are achieved [8-10]. The absence of recurrence and preservation of splenic tissue on follow-up visits in our patient parallels outcomes reported in recent pediatric series.

Alternative techniques such as cyst deroofing, marsupialization, or percutaneous drainage have been described, particularly for large cysts; however, multiple studies report higher recurrence rates due to persistence of the epithelial lining [9]. In contrast, although with greater technical demands, partial splenectomy offers definitive treatment while maintaining immunologic protection. The successful laparoscopic management in this case highlights that, with meticulous vascular control, dissection, and appropriate patient selection, minimally invasive partial splenectomy is feasible even for giant cysts in children.

Overall, this case supports growing evidence that laparoscopic partial splenectomy represents an optimal balance between definitive cyst management and splenic preservation. Early surgical intervention in symptomatic pediatric patients may prevent potentially life-threatening complications while ensuring excellent long-term functional outcomes.

## Conclusions

Giant congenital splenic cysts are uncommon but may cause significant symptoms due to mass effect. Laparoscopic partial splenectomy provides a safe and effective treatment option while preserving splenic immunologic function in pediatric patients. Early evaluation and timely surgical management help prevent potential complications, ensure durable symptom relief, and support full recovery. Continued clinical experience and long-term follow-up will further clarify optimal treatment strategies for these rare lesions.
